# PReS13-SPK-1577: 'Asia' - autoimmune (auto-inflammatory) syndromes induced by adjuvants

**DOI:** 10.1186/1546-0096-11-S2-I28

**Published:** 2013-12-05

**Authors:** YY Shoenfeld

**Affiliations:** 1 Medicien B, Sheba Medical Center, Tel Hashomer, Israel

## 

In the last decades four enigmatic medical conditions were described, all of which were characterized by an hyperactive immune response as well as similar clinical and laboratory manifestations. These conditions, namely siliconosis, the Gulf war syndrome (GWS), the macrophagic myofasciitis syndrome (MMF) and post vaccination phenomena represent environmental factors that may play a role in inducing or aggravating autoimmunity and auto-inflammation.

Vaccines, administered to animal and humans, are clearly one of the best achievements of modern medicine and are commonly and safely inoculated to the vast majority of subjects. However in rare occasions vaccines may induce autoimmune or auto-inflammatory conditions both in animals and in humans. These conditions, either defined diseases such as Gullian Barre syndrome or enigmatic ones, have been reported following different vaccines and vaccination protocols. The susceptibility factors and the temporal association between vaccines and these rare immune mediated reactions are yet to be defined, however the similarities between vaccines and infections and the addition of an adjuvant (i.e. alum, squalene etc.) to almost every vaccine are considered major contributors to such adverse events. Perhaps the most evaluated condition is MMF, in which a cause was clearly delineated. MMF is a rare condition caused by deposition of alum, used to adjuvant different vaccines, which bring about an immune mediated muscles disease. Thus, in only a minority of genetically prone, i.e. HLA-DRB1*01 patients, alum may induce this syndrome.

Another immune mediated phenomena leading to autoimmune diseases is exposure to silicones (i.e. breast implant). In a large study Fryzek *et a.l * showed that a group of 1546 patients with silicone breast implants had a statistically significant increase in 16 of 28 investigated symptoms when compared to a group of 2496 women who underwent reduction mammoplasties. Many of these symptoms satisfied several criteria for fibromyalgia and chronic fatigue syndrome, which is congruent with the FDA's finding that there is a statistically significant link between fibromyalgia and ruptured silicone gel implants.

A common denominator to each of these four syndromes as well as to various infectious agents is the trigger entailing adjuvant activity (7).

Thus, herein we would like to suggest to include these four conditions under a common syndrome entitled the "Autoimmune (Auto-inflammatory) Syndrome induced by adjuvants" (ASIA). We propose several major and minor criteria that may aid in the diagnosis of this newly defined condition (ASIA).

## Disclosure of interest

None declared.

